# Morphometric Analysis of Brain in Newborn with Congenital Diaphragmatic Hernia

**DOI:** 10.3390/brainsci11040455

**Published:** 2021-04-02

**Authors:** Martina Lucignani, Daniela Longo, Elena Fontana, Maria Camilla Rossi-Espagnet, Giulia Lucignani, Sara Savelli, Stefano Bascetta, Stefania Sgrò, Francesco Morini, Paola Giliberti, Antonio Napolitano

**Affiliations:** 1 Medical Physics Department, Bambino Gesù Children’s Hospital, IRCCS, 00165 Rome, Italy; martina.lucignani@opbg.net; 2 Neuroradiology Unit, Imaging Department, Bambino Gesù Children’s Hospital, IRCCS, 00165 Rome, Italy; daniela.longo@opbg.net (D.L.); elena.fontana@opbg.net (E.F.); mcamilla.rossi@opbg.net (M.C.R.-E.); giulia.lucignani@opbg.net (G.L.); 3 NESMOS Department, Sant’Andrea Hospital, Sapienza University, 00189 Rome, Italy; 4 Imaging Department, Bambino Gesù Children’s Hospital and Research Institute, 00165 Rome, Italy; sara.savelli@opbg.net (S.S.); stefano.bascetta@opbg.net (S.B.); 5 Department of Anesthesia and Critical Care, Bambino Gesù Children’s Hospital, IRCCS, 00165 Rome, Italy; stefania.sgro@opbg.net; 6 Department of Medical and Surgical Neonatology, Bambino Gesù Children’s Hospital, IRCCS, 00165 Rome, Italy; francesco.morini@opbg.net (F.M.); paola.giliberti@opbg.net (P.G.)

**Keywords:** magnetic resonance imaging (MRI), congenital diaphragmatic hernia (CDH), cortical thickness (CT), local gyrification index (LGI), total lung volume (TLV)

## Abstract

Congenital diaphragmatic hernia (CDH) is a severe pediatric disorder with herniation of abdominal viscera into the thoracic cavity. Since neurodevelopmental impairment constitutes a common outcome, we performed morphometric magnetic resonance imaging (MRI) analysis on CDH infants to investigate cortical parameters such as cortical thickness (CT) and local gyrification index (LGI). By assessing CT and LGI distributions and their correlations with variables which might have an impact on oxygen delivery (total lung volume, TLV), we aimed to detect how altered perfusion affects cortical development in CDH. A group of CDH patients received both prenatal (i.e., fetal stage) and postnatal MRI. From postnatal high-resolution T2-weighted images, mean CT and LGI distributions of 16 CDH were computed and statistically compared to those of 13 controls. Moreover, TLV measures obtained from fetal MRI were further correlated to LGI. Compared to controls, CDH infants exhibited areas of hypogiria within bilateral fronto-temporo-parietal labels, while no differences were found for CT. LGI significantly correlated with TLV within bilateral temporal lobes and left frontal lobe, involving language- and auditory-related brain areas. Although the causes of neurodevelopmental impairment in CDH are still unclear, our results may suggest their link with altered cortical maturation and possible impaired oxygen perfusion.

## 1. Introduction

Congenital diaphragmatic hernia (CDH) is a severe pediatric disorder characterized by herniation of abdominal viscera into the thoracic cavity through a diaphragmatic defect, mostly on the left side [1], associated with a variable degree of pulmonary hypoplasia, pulmonary hypertension, and cardiac function abnormalities. The defect occurs in the first gestational weeks as a consequence of altered diaphragm embryogenesis, and may present with a broad spectrum of severity. CDH is commonly diagnosed at prenatal ultrasound (US) during the second trimester of pregnancy and surgically treated during the first weeks after birth. In the last few decades, the use of extracorporeal membrane oxygenation (ECMO) and advances in the neonatal surgical field have improved the rate of survival even in more severely affected children. CDH survivors present a wide variety of long term morbidity, ranging from respiratory to orthopedic problems [2]. Among these, long-term neurological sequelae raise particular concern [3], although studies describing the neurological outcome of CDH survivors who are not treated with neonatal ECMO are limited [4]. Earlier studies report long-term outcomes, with evidence of impaired language and visual motor skills, behavioral, cognitive and developmental delay and hearing loss [5,6,7,8,9,10]. This evidence was further confirmed for short-term evaluation, where neurodevelopmental assessment at one year of age revealed mild (44%) and severe (13%) delay in at least one domain among cognitive, language and motor functions, depending on several determinants such as intubation, oxygen requirement and intensive care unit stay [4,11]. Although neurological sequelae have been frequently described, the timing and mechanism of brain injury remain poorly understood [12]. Magnetic resonance imaging (MRI) allows us to investigate structural neuropathological lesions in CDH infants, revealing brain and parenchymal injuries [13,14], with ventricular enlargement and intracranial hemorrhage appearing already at the fetal stage [14,15,16]. Among MRI analysis, morphometric techniques could represent powerful tools as they allow investigating specific cortical parameters usually implicated in several neurodevelopmental disorders, thus moving a further step toward a better understanding of brain injury mechanisms. A morphometric approach was recently proposed to measure brain structures of CDH fetuses in terms of fronto-occipital, brain biparietal and bone biparietal diameters, revealing enlarged extra-axial spaces and cerebellar dimensions associated with CDH severity [16]. In this context, we proposed a morphometric assessment to investigate subtle cortical alterations of cortical thickness (CT) and local gyrification index (LGI) on CDH infant brains, since their role in neurological development has been fully addressed [17,18]. CT represents a sensitive indicator of normal brain structural and functional development [19,20], aging, as well as a variety of neuropsychiatric disorders. Normally, CT varies between 1 and 4.5 mm with an average value of approximately 2.5 mm [21], while abnormalities are commonly observed in neuro-developmental disease, including schizophrenia, autism [22] and bipolar disorders [23]. LGI quantifies regional pattern of gyrification, playing a role in the normal brain development [24]; it changes with age and it may be an indicator of cognitive functions [24,25,26].

The total lung volume (TLV) was also estimated from fetal MRI as a possible indirect measure of oxygen deliver and perfusion and it was used in our study to properly stratify the severity of the CDH cases and to observe possible correlation with postnatal cortical parameters. By evaluating cortical parameter (CT and LGI) distributions and assessing their correlation with TLV, we aimed to detect altered brain areas and to determine how altered oxygen delivery may affect the cortical development process.

## 2. Materials and Methods

### 2.1. Subjects

This is an observational, case-control, cross-sectional study of data collected from prenatally diagnosed CDH patients. The study protocol was in agreement with the principles outlined in the Declaration of Helsinki and was approved by the ethical committee. Written informed consent was obtained from every parent/guardian of neonatal subject for the execution of the MR exams. Between November 2015 and April 2019, 27 CDH neonates were admitted to the Bambino Gesù Children’s Hospital neonatal intensive care unit (NICU). Out of 27 subjects, 24 received the acquisition protocol defined in this study. Patients with chromosomal abnormalities, major cardiovascular anomalies, kidney and cerebral defects and those who required ECMO were excluded from the study. Based on these exclusion criteria, we identified 22 neonates for our investigation. Additionally, a group of healthy controls (HC) with no neurologic disorder and unremarkable brain MRI was included in the study and served as controls. In particular, HC were included if they had negative neurologic examination and underwent MRI for non-neurologic conditions including cystic lymphangioma, esophageal atresia or facial vascular malformations without intracranial component.

### 2.2. Magnetic Resonance Imaging (MRI) Acquisition

Twenty two CDH infants and 13 HC, fulfilling inclusion criteria, underwent brain MRI within the first two months of life. All patients were scanned without sedation using a feed and wrap protocol [27,28] with ear protection and proper immobilization system (MedVac infant full body splint). Image protocol included: 3D magnetization-prepared rapid acquisition with gradient echo (MPRAGE) (TR/TE = 2060/2.2 ms, FA = 9°, ST = 0.8 mm) and TSE T2-weighted sequence (TR/TE = 3000/400 ms, FA = 120°, ST = 0.9 mm), axial TSE T2 weighted sequence (TR/TE = 6380/108 ms, FA = 150°,ST = 3 mm), axial gradient echo (GE) or susceptibility weighted imaging (SWI) sequence (TR/TE = 27/20 ms, FA = 15°, ST = 1.5 mm), axial diffusion weighted imaging (DWI) (TR/TE = 9000/98 ms, FA = 90°, ST = 3 mm) and diffusion tensor imaging (DTI) (TR/TE = 7900/95 ms, FA = 90°, ST = 2 mm). All images were acquired on a 3T Siemens Magnetom Skyra scanner (Siemens, Erlangen, Germany) equipped with 32 channels head-coil (coil dimensions L-W-H: 440 mm × 330 mm × 370 mm). Among the 22 subjects identified, 12 had also been prenatally scanned (i.e., at fetal stage) according to the protocol defined by Savelli et al. [29].

### 2.3. Image Processing

Immature myelination of the white matter (WM) in neonatal brains results in inverted MRI contrast [30], thus requiring image preprocessing to be performed on 3D T2-weighted turbo spin echo sequences rather than T1-weighted structural MRI [31]. From volumetric T2-weighted images, 2 neuroradiologists evaluated brain injury for each patient, using the scoring system of Radhakrishnan et al. [32]. A neonatal specific processing pipeline was exploited to tackle the peculiar characteristics of neonatal brain images (e.g., motion artefacts, inverted WM–grey matter (GM) contrast, low resolution, low signal-to-noise ratio, partial volume effects). Data were processed through a dedicated algorithm, consisting of a combination of voxel-based and surface-based morphometric techniques. An automatic pipeline sequentially performed skull-stripping, noise, bias and intensity inhomogeneity correction to further produce voxel-based segmentation, i.e., the classification of the brain image in its main tissues (WM, GM and cerebro-spinal fluid, CSF). We performed voxel-based segmentation by properly adapting the Computational Anatomy Toolbox (C.Gaser, Structural Brain Mapping Group, Jena University Hospital, Jena, Germany; http://dbm.neuro.uni-jena.de/cat/, accessed on October 2017) [33] pipeline to work with the inverted WM/GM contrast typical of infant data. Brain segmentation was attained based on the image intensity as well as tissue class priors which indicate the likelihood of finding a given tissue class at a given location. Once segmented volumes were obtained, the pipeline provided cortical surfaces reconstruction by means of FreeSurfer software (http://surfer.nmr.harvard.edu/, accessed on April 2016), the gold standard surface-based method for MR image processing. The WM/GM boundary was first determined as the edge of the segmented WM and then tessellated to generate the inner cortical surface (white surface), while the outer surface (pial surface) was generated through the expansion of white surface with a point-to-point correspondence. According to the approach described by Fischl et al., CT was computed as the average of the distance measured from each surface to the other [34]. LGI was computed using the novel approach proposed by Lyu et al. (https://github.com/ilwoolyu/LocalGyrificationIndex, accessed on November 2019) that quantifies cortical gyrification within sulcal and gyral regions using a spatially varying kernel shape, able to adaptively encode cortical folding patterns. The proposed LGI is then computed within the adaptive kernel as a ratio of the cortical surface area and a fixed area on the outer hull (ρ = 316 mm^2^) [35,36]. Finally, the TLV was calculated using SYNGOVia software as the sum of the manually identified lung volumes of the contra-lateral lung and, when visible, of the ipsi-lateral lung compressed by the presence of the herniated viscera in the thorax [27]. For statistical purposes and visualization, we resampled cortical parameters onto a common surface template provided by the University of North Carolina and representing a two-month-old brain atlas.

### 2.4. Statistical Analysis

We investigated differences in cortical parameters distributions among groups. To this end, we mapped vertex-wise CT and LGI values on a common spherical coordinate system using spherical transformation. We assessed differences among groups using permutation tests (1000 permutations for all tests) based on the *t* statistics, performed with the Permutation Analysis of Linear Models (PALM) FMRIB software library (FSL) package. Particularly, we used group age as covariate to produce threshold-free cluster enhancement (TFCE) statistical maps, where the initial raw statistical images were enhanced using both the intensity of the data point and information from neighboring voxels [37]. We detected group differences on family-wise error (FWE) corrected *p*-value maps. Moreover, correlation analyses were evaluated vertex-wise between cortical parameters (CT and LGI) and the TLV clinical variable, testing Pearson correlation with PALM permutation test (1000 permutations).

## 3. Results

Among 22 CDH fulfilling the inclusion criteria, 6 subjects were excluded from the statistical analysis due to processing issues related to image artefacts. Consequently, statistics was focused on the remaining 16 CDH patients (mean gestational age at birth = 38 weeks, mean age at postnatal MRI = 5.6 weeks, age range = 3–9 weeks, 56% female) and 13 age- and gender-matched HC. Demographic data of both CDH and HC are reported in Table 1, while clinical and radiological data of CDH patients are summarized in Table 2 and Table 3. CDH vs. HC did not show any differences in age (t = 0.141, *p* = 0.889) and gender (χ^2^ = 0.083; *p* = 0.774). Since fetal MRI was not available for all CDH, TLV values were reported only for 12 patients.

### 3.1. Conventional MRI Findings

**Figure 1:**
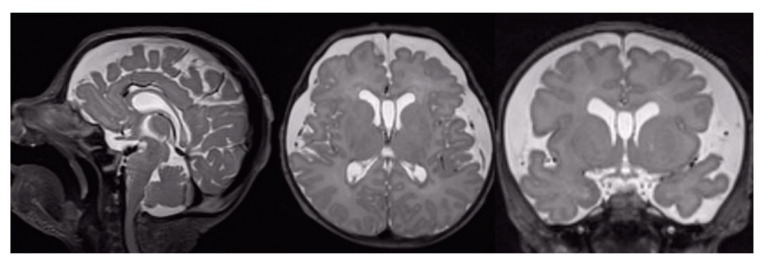
T2-weighted MRI of a CDH patient. Sagittal, axial and coronal view of the brain shows an enlargement of the SAS in the fronto-insular region bilaterally.

The evaluation of conventional MR images demonstrated the presence of positive findings (damage score greater than 0) in 10/16 patients (62.5%). In particular, the following were documented: ventriculomegaly in 4 patients (24.8%), increased subarachnoid spaces (SAS) in 6 patients (37.5%), intraventricular haemorrhage in 3 patients (18.7%), intraparenchymal haemorrhage in 3 patients (18.7%), cerebellar haemorrhage in 1 patient (6.2%), WM damage in 1 patient (6.2%), GM damage in 1 patient (6.2%), basal ganglia damage in 2 patients (12.5%) The increase of the SAS (Figure 1) was the most frequently found finding in the sample of our patients (Table 4).

### 3.2. Cortical Thickness Results

Mean distribution of CT was computed for both hemispheres (left hemisphere LH and right hemisphere RH) in CDH (CTLH = 2.13 ± 0.29 mm, CTRH = 2.11 ± 0.32 mm) and HC (CTLH = 2.17 ± 0.33 mm, CTRH = 2.17 ± 0.34 mm). No significant differences were found between CT distribution of CDH patients and HC.

### 3.3. Gyrification Results

**Figure 2:**
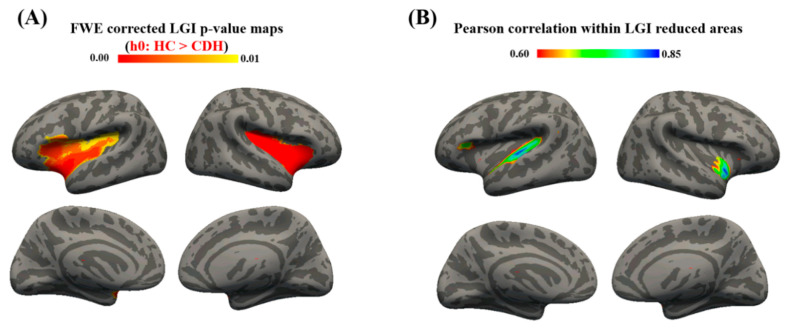
Statistical results mapped on the inflated surface of a 2-months template. (**A**) Family-wise error (FWE) corrected p-value maps for LGI comparison, obtained for HC > CDH contrast. Significance was set at 0.01. (**B**) Brain areas with significant total lung volume-local gyrification index (TLV-LGI) correlation and significant reduced gyrification.

Mean distribution of LGI was computed for both hemisphere in CDH (LGILH = 2.52 ± 0.74, LGIRH = 2.56 ± 0.83) and HC (LGILH = 2.65 ± 0.93, LGIRH = 2.67 ± 1.03). CDH showed several brain areas with significant reduction in gyrification (i.e., hypogyria) when compared to HC, while no regions presented increased LGI (Table 5). As reported in Figure 2A, the reduced gyrification was mainly located within bilateral frontal (pLH = 0.007, pRH = 0.003), parietal (pLH = 0.009, pRH = 0.002) and temporal lobes (pLH = 0.008, pRH = 0.001).

### 3.4. Correlation Results

**Figure 3:**
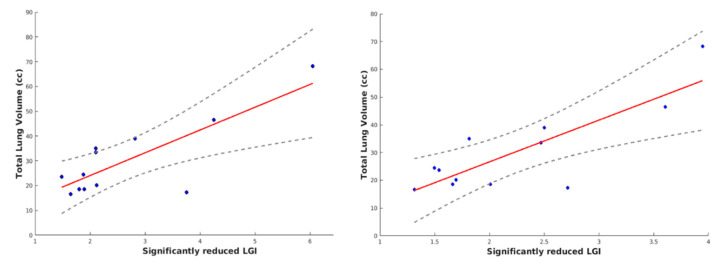
Scatter plot of TLV-LGI correlation for LH (left) and RH (right). Scatter plot with line of best fit (red line) within 95% confident interval (dashed grey lines).

We computed TLV from fetal MRI of 12 CDH patients (Table 3) and then assessed its vertex-wise correlation with cortical parameters like CT and LGI. Pearson analysis revealed no significant correlation between CT and TLV in all cortical areas analyzed, while LGI-TLV correlation was found within temporal lobes and left frontal lobe bilaterally. Focusing on brain areas where LGI was both significantly reduced and correlated with TLV (Figure 2B), we found slightly reduced significant clusters within the left frontal lobe and bilateral temporal lobes (Table 6). Averaging significantly reduced LGI, it was possible to produce a TLV-LGI correlation scatter plot (Figure 3).

## 4. Discussion

To the best of our knowledge, this is the first study that attempted to quantify subtle cortical alteration in CDH survivors as compared to age-matched HC by exploring cortical thickness and gyrification. Concerning LGI, the novel approach proposed by Lyu et al., allowed us to accurately detect morphological alterations at the scale of a single gyrus/sulcus as demonstrated in other studies [36]. The ability of revealing subtle gyrification distribution within single-gyrus cluster produced sharper and more detailed results, especially on neonatal brain surfaces [35]. We observed no cortical thinning or thickening, but significant gyrification reduction (hypogyria) within parietal lobes, extending to frontal and temporal lobes. Firstly, the presence of these symmetrically distributed hypogyria areas suggests altered volumetric growth and maturation as it has been already seen in other postnatal CDH neuroimaging studies [12,13,15,32]. In particular, such altered maturation was actually assumed as the results of reduced myelination, cortical infolding [12] and delayed sulcation [14] occurring in CDH infants. Neuroimaging investigations had already shown evidence of brain injury, including parenchymal abnormalities and enlarged extra-axial spaces as the most common findings, usually attributed to delayed brain maturation [38], but no evidence of subtle alterations of cortical parameters has been reported so far. In this context, the proposed morphometric approach seems to provide additional details on microstructural brain injury, allowing us to detect vertex-wise cortical parameter distribution. Although brain abnormalities were well addressed in CDH infants, brain growth and cortical alteration occurring in utero still remain unclear. Tracy et al. reported abnormal prenatal neuroimaging findings in only 4 CDH patients, one of which had an abnormal cortical gyration pattern [15]. Recently, both traditional [14] and morphological [16] MRI investigation were performed on CDH fetuses, revealing associations between prenatal and postnatal enlarged extra-axial spaces, but an absence of delayed sulcation and parenchymal abnormalities in fetuses. Since brain parenchymal injury was absent in fetuses and appeared in the postnatal stage, they hypothesized that cortical alteration might be related to postnatal factors such as NICU stay, ECMO or surgery [16]. Conversely, it is possible that enlarged extra-axial spaces found in prenatal stage may be attributed to cortical development alteration that takes place in utero as demonstrated for fetuses with complex heart disease [38], but this hypothesis has yet to be tested for CDH. Most likely, the missing evidences of fetal parenchymal modifications were probably related to the sensitivity of the investigation methods, which were not able to detect subtle brain abnormalities. Furthermore, such findings, however subtle, might be concomitant with cortical folding itself [39] and then be indicative of dormant and slightly visible abnormal development.

A close link exists between cortical structural and functional organization, as it has been seen in developmental and neuropsychiatric disorders where modifications in gyral and sulcal distribution were responsible for neurological outcome [40,41,42]. Early brain development alteration might occur long before neurological symptoms and consequently they could represent a first marker for future neurodevelopmental impairments [43]. In patients with CDH, MRI findings of postnatal brain injury were associated with motor dysfunction [15], lower cognitive scores and language deficits [9,10,12], but no correlation resulted between neurological outcome and prenatal MRI findings [14]. These findings were further confirmed for long-term outcomes, resulting in impaired language and visual motor skills, behavioral, cognitive, developmental delay and hearing loss. The vertex-wise morphometric approach performed in this study highlights some cortical areas with hypogyria. In particular, LGI was significantly reduced in canonical language-related areas (pars opercularis and pars triangularis within Broca’s area, while superior temporal in Wernicke’s area) and in auditory-related areas (located within transverse temporal labels). As it was demonstrated for schizophrenic subjects [44,45], we assumed that alteration in cortical parameters within language- and auditory-related areas could be responsible for corresponding language and auditory long-term neurological deficits. Since the severity and outcome of CDH patients depends from lung volume, liver herniation and lung-to-head ratio [46,47,48,49], we might hypothesize a similar prognostic role for altered cortical morphometry. Based on recent findings revealing significant association of lung hypoplasia severity with postnatal brain injury in CDH [14], we postulated that reduced lung volumes could be associated with altered brain oxygenation, that in turn might lead to a delayed cortical development. Consequently, investigating vertex-wise correlation between LGI and TLV, we observed significantly correlated areas located within left frontal lobe (pars opercularis, pars traingularis) and bilateral temporal lobes (superior temporal). Focusing on clusters where LGI was both significantly reduced and correlated with TLV, we identified three main areas within left brain, e.g., pars opercularis, pars triangularis and superior temporal (respectively, Brodmann areas 44, 45 and 22), normally implicated in language production and comprehension. Also, superior temporal areas within the right brain (Brodmann area 38) participate in several language functions, including semantic processing and speech comprehension. These findings are in agreement with neurological impairment described in long- and short-term studies on CDH.

There might be also interest in investigating similarities and differences of CDH and other infant pathologies leading to similar neurodevelopmental deficits, specifically focusing on pathologies that are characterized by a reduced oxygen deliver e.g., congenital heart disease (CHD) and preterm infants [7]. Also CHD and preterm infants exhibit brain lesions due to reduced oxygen perfusion, respectively caused by heart malformations and bronchopulmonary dysplasia. There is very little evidence in the literature about the evaluation of cortical parameters in CHD and preterm infants, as most of these studies investigated the cortex only during their childhood/adolescence [50,51,52,53]. Claessens et al. found cortical alteration in neonates with severe CHD, in terms of postoperative cortical GM (CGM) volumetric growth and maturation, that appeared significantly reduced when compared to HC. Conversely, no differences were recorded in thickness and LGI distribution. CGM reduction has driven the hypothesis that hypoxia conditions and possible inflammation are a consequences of invasive surgery [54]. In fact, brain inflammation influences the development of pre-oligodendrocytes, i.e., fetal WM cells that play an important role in driving cortical development, which fail to myelinate the developing axons under hypoxic conditions, thus leading to abnormal cortical maturation [55]. In contrast with unaltered LGI distribution within CHD population found by Claessens, animal studies have shown significant effects of hypoxia on cortical gyrification [56,57], that was further confirmed by a recent study on CHD newborns showing reduced cortical volume and gyrification within temporal, parietal and occipital lobes [58]. Among CHD, studies on hypoplastic left heart syndrome (HLHS) revealed altered cortical development both in fetuses [59] and in infants [60]. Brain hypoperfusion related to the reduced outflow from the left heart in HLHS have negative effects on brain maturation, leading to lower gyrification indices in the frontal, temporal, cingulate, postcentral, calcarine, occipital, collateral, sylvian and parieto-occipital areas of HLHS fetuses [59]. Our results of apparent reduced cortical maturation in terms of LGI within areas of bilateral parietal, frontal and temporal lobes partially overlap with previous studies on CHD and could, therefore, be explained as a consequence of reduced oxygenation, since also CDH neonates are exposed to the potential risk of hypoxia [7]. Brain development delay and corresponding neurobehavioral impairment was also seen in preterm infants when compared to term-born neonates (Huppi et al., 1996). Although the hypothesis of an impaired maturation could suggest a parallelism of CDH with the preterm condition, the heterogeneous cortical development results on preterm subjects are not helpful for drawing any conclusion. In fact, some studies found that preterm infants have larger CGM volumes compared to HC [61,62], while others found smaller volumes [63]. A study investigating thickness and gyrification on preterm infants imaged at 30 and 40 weeks postmenstrual age (PMA) revealed increased median CT and decreasing LGI for increasing brain abnormality score, computed in terms of unmyelinated WM, cortical and deep GM, cerebellar and total brain abnormalities detected with visual inspection and 2D measurements [64]. Despite the accordance between our study and Moeskops’ in finding reduced LGI, we should be aware about the different study setting. While we compare groups based on their pathological nature (CDH or healthy subjects), Moeskops evaluated cortical differences in preterm infants classified for different brain abnormality score (normal, mild, moderate and severe at both 30 and 40 PMA). Furthermore, ex-preterm children, adolescents and adults show abnormal cortical patterns when compared with their control counterparts. Different studies agree in finding regionally thicker and thinner cortical regions, where the thickening process mainly took place within the frontal lobes bilaterally, while thinning appeared predominately within the left temporal lobes [52,65]. Moreover, recent CT assessment on children born extremely preterm (EPT) revealed altered distribution within language brain areas, where resulted increased thickness for intra cranial volume (ICV) normalized data [53]. Cortical folding assessment resulted in patterns of increased bilateral temporal lobes gyrification in preterm children [66] and patterns of significantly reduced LGI in extensive cortical regions of frontal, anterior temporal, and occipito-parietal lobes in adults [50]. This evidence is partially in contrast with our finding of absence of CT alteration and reduced LGI, thus the hypothesis that the two conditions may not share the same dysfunction path seems to be plausible, but the different age ranges of the subjects studied must be considered. Although our study provides interesting evidence of altered morphometry within areas commonly involved in the neurological impairment in CDH, there are a few limitations to consider. First, only a subset of patients fulfilling our inclusion criteria were pooled in the analysis. The number resulted indeed in being relatively small since several CDH subjects required ECMO treatment and needed to be excluded. Moreover, since fetal MRI investigations were partially conducted in other institutes, prenatal variables such as TLV were not available for all subjects, thus representing a limitation for the generalizability of the results as correlation might be prejudiced by the small subject number. A validation study on more subjects (both at fetal and neonatal stage) needs to be conducted in the future to avoid any over interpretation of the results. Our clinically heterogeneous dataset (i.e., possible different modes of ventilation, blood transfusion, sepsis rate, structural heart defects) does not allow us to draw clear and specific conclusions on the time that the injury occurs but, unfortunately, the clinical path taken by each patients strongly depends on the complexity of the disorder. On the other hand, a hypothesis of an earlier altered brain growth at the prenatal stage would fit with the idea that these cortical folding changes might require long time to establish and the timing between birth and postnatal MRI might not be sufficient for it.

## 5. Conclusions

In conclusion, for the first time morphometric analysis was applied to investigate subtle cortical alteration in CDH newborns, finding significant reduced cortical maturation in extended brain areas. In addition, a correlation was found between a prenatal predictor of CDH severity (namely TLV) and LGI. These data, together with the role of specific clinical variables, may be useful in predicting the short- and long-term neurological outcome in CDH survivors. Future studies are needed to extend the morphometric analysis on fetuses in order to highlight timing of cortical development alteration, to explore a possible association between CDH severity indicators and brain morphometric parameters, and to evaluate the impact of brain morphometric parameters on long-term neurological outcomes.

## Figures and Tables

**Table 1:** Demographic data of congenital diaphragmatic hernia (CDH) and healthy controls (HC).

	CDH	HC
Age		
Mean	5.52	5.41
SD	1.80	1.94
Range	3.1–9.4	3.0–9.0
Gender		
Female (%)	9 (56%)	8 (61%)
Male (%)	7 (44%)	5 (39%)

**Table 2:** Clinical data of CDH patients.

CDH	Gender	Herniation Side	Surgery (Day after Birth)	Days of Ventilation	Structural Heart Defect	# Sepsis	Units of Blood Transfused
sub-01	M	L	3	8	N	0	0
sub-02	F	L	2	9	N	0	0
sub-03	F	L	6	11	PDA	2	4
sub-04	F	L	2	7	PDA	0	0
sub-05	F	L	4	11	N	1	1
sub-06	M	L	2	10	ASD+PDA	0	0
sub-07	F	L	3	9	PDA+PFO	0	1
sub-08	M	L	125	18	ASD	0	2
sub-09	M	L	4	7	PDA	0	0
sub-10	F	L	40	48	ASD+PFO	1	1
sub-11	F	L	54	11	ASD+PDA	1	1
sub-12	M	L	2	13	PDA	0	1
sub-13	F	L	2	19	PDA	0	1
sub-14	M	L	3	24	ASD+PDA	0	1
sub-15	F	R	3	11	PDA	0	1
sub-16	M	L	6	19	PDA+PH	0	3

N = Nothing; PDA = patent ductus arteriosus; PFO = patent foramen ovale; ASD = atrial septal defect; PH = pulmonary hypertension; # Sepsis = number of sepsis.

**Table 3:** Radiological data of CDH patients.

CDH	Gestational Age at Fetal MRI (weeks)	Age at Postnatal MRI (weeks)	Ipsi-Lateral Lung Vol (cc)	Contra-Lateral Lung Vol (cc)	Total Lung Volume (cc)
sub-01	31	4.4	6.0	33.0	39.0
sub-02	30	7.3	N.D.	16.6	16.6
sub-03	31	6	N.D.	17.3	17.3
sub-04	31	7.4	N.D.	20.1	20.1
sub-05	28	3.3	1.5	17.0	18.5
sub-06	--	4.1	--	--	--
sub-07	32	4.3	9.9	36.6	46.5
sub-08	--	4.7	--	--	--
sub-09	--	5	--	--	--
sub-10	29	6.6	N.D.	33.5	33.5
sub-11	32	9.4	1.5	17.0	18.5
sub-12	37	3.4	N.D.	24.5	24.5
sub-13	29	4.9	7.0	28.0	35.5
sub-14	33	8.1	16.3	52.0	68.3
sub-15	--	6.4	--	--	--
sub-16	30	3.1	N.D.	23.6	23.6

N.D. = Not Detectable from fetal magnetic resonance imaging (MRI).

**Table 4:** Brain injuries in CDH patients at postnatal MRI.

Brain Damage	CDH Patients at Postnatal MRI
Ventriculomegaly	4 (24.8%)
SAS enlargement	6 (37.5%)
Intraventricular hemorrhage	3 (18.7%)
Intraparenchymal hemorrhage	3 (18.7%)
WM damage	1 (6.2%)
GM damage	1 (6.2%)
Basal ganglia damage	2 (12.5%)

**Table 5:** Areas of significantly reduced LGI. For each cortical lobe where CDH patients showed significantly reduced LGI compared to HC, we reported mean gyrification values for HC and CDH, mean *p*-value and list of cortical labels involved, sorted by the number of significant elements within the cluster (increasing cluster extent).

	HC Mean (sd)	CDH Mean (sd)	*p*-Value	Main Involved Structures (Ordered by Cluster Extent)
***Left Brain***				
Frontal lobe	3.29 (0.75)	2.13 (0.34)	0.007	Pars Opercularis, Lateral Orbito Frontal, Pars Triangularis, Pre-central
Parietal lobe	4.62 (0.52)	2.94 (0.22)	0.009	Supramarginal, Post-central, Inferior Parietal
Temporal lobe	3.75 (0.63)	2.30 (0.38)	0.008	Insula, Superior Temporal, Transverse Temporal, Banks-sts, Middle Temporal, Temporal Pole
***Right Brain***				
Frontal lobe	4.25 (0.32)	2.88 (0.22)	0.003	Lateral Orbito Frontal, Pars Triangularis, Pre-central, Pars Orbitalis
Parietal lobe	6.16 (0.68)	3.87 (0.46)	0.002	Supramarginal, Post-central
Temporal lobe	5.56 (1.17)	3.28 (0.74)	0.001	Insula, Superior Temporal, Transverse Temporal

**Table 6:** Areas of significant TLV-LGI correlation. For each cortical lobe, we identified areas exhibiting both conditions of significantly reduced LGI and TLV-LGI correlation; main involved areas, mean CDH and HC gyrification and mean correlation were reported, together with their *p*-value.

	LGI CDH	LGI HC (*p*-Value)	Correlation (*p*-Value)	Main Involved Structures
***Left Brain***				
Frontal lobe	1.91	2.75 (0.006)	0.72 (0.04)	Pars Opercularis, Pars Triangularis
Temporal lobe	2.54	3.99 (0.007)	0.73 (0.03)	Superior Temporal, Transverse Temporal
***Right Brain***				
Temporal lobe	2.26	4.27 (0.001)	0.76 (0.03)	Insula, Superior Temporal

## Data Availability

The data presented in this study are available on request from the corresponding author. The data are not publicly available due to privacy and ethical restrictions.
